# A Novel Cardiotoxic Mechanism for a Pervasive Global Pollutant

**DOI:** 10.1038/srep41476

**Published:** 2017-01-31

**Authors:** Fabien Brette, Holly A. Shiels, Gina L. J. Galli, Caroline Cros, John P. Incardona, Nathaniel L. Scholz, Barbara A. Block

**Affiliations:** 1 Stanford University, Biology Department, Hopkins Marine Station, Pacific Grove, CA, 93950, USA; 2 Division of Cardiovascular Sciences, Faculty of Biology, Medicine and Health, University of Manchester, Manchester, M13 9NT, UK; 3 National Oceanic and Atmospheric Administration, Northwest Fisheries Science Center, Seattle, Washington, 98112, USA; 4 Tuna Research and Conservation Center, Stanford University Hopkins Marine Station, Pacific Grove, CA, 93950, USA.

## Abstract

The Deepwater Horizon disaster drew global attention to the toxicity of crude oil and the potential for adverse health effects amongst marine life and spill responders in the northern Gulf of Mexico. The blowout released complex mixtures of polycyclic aromatic hydrocarbons (PAHs) into critical pelagic spawning habitats for tunas, billfishes, and other ecologically important top predators. Crude oil disrupts cardiac function and has been associated with heart malformations in developing fish. However, the precise identity of cardiotoxic PAHs, and the mechanisms underlying contractile dysfunction are not known. Here we show that phenanthrene, a PAH with a benzene 3-ring structure, is the key moiety disrupting the physiology of heart muscle cells. Phenanthrene is a ubiquitous pollutant in water and air, and the cellular targets for this compound are highly conserved across vertebrates. Our findings therefore suggest that phenanthrene may be a major worldwide cause of vertebrate cardiac dysfunction.

Polycyclic aromatic hydrocarbons (PAHs), consisting of two or more fused benzene rings, are ubiquitous pollutants in aquatic systems. Crude oils typically produce PAH mixtures with high concentrations of 2-, 3-, and 4-ringed PAHs families and correspondingly low levels of ≥5-ring compounds, while combustion of fossil fuels and other carbon sources generate mixtures that may be dominated by larger 4–6 ring compounds. Although industrial PAH emissions have declined in the developed world, loadings to aquatic habitats have increased with human population growth and increased motor vehicle use^1^,^2^. Chronic aquatic PAH loading occurs by multiple pathways, including airborne deposition of soot and exhaust particles^1^,^3^, runoff from impervious surfaces^4^, and direct absorption of vapor-phase compounds from the air^5^. By contrast, catastrophic oil spills such as the 1989 Exxon Valdez tanker grounding^6^ and the 2010 Deepwater Horizon wellhead blowout released very large quantities of PAHs directly into the marine environment^7^. Certain heterocyclics contain nitrogen, sulphur, or oxygen, and are more technically termed polycyclic aromatic compounds (PACs). However, following general convention, we refer to heterocyclics here as PAHs.

As expected for a large and complex family of structurally related compounds, the biological activities of PAHs are both overlapping and multifaceted. A major challenge has been determining which individual compounds contribute to the overall toxicity of complex mixtures with different chemical compositions. The carcinogenicity of 4–6-ringed PAHs such as benzo(a)pyrene was recognized more than half a century ago^8^ and occurs in both terrestrial and aquatic organisms. However, the cardiotoxicity of 3-ringed PAHs was not suspected until the aftermath of the 1989 Exxon Valdez disaster when studies documented developmental defects and mortality in fish embryos exposed to Alaskan North Slope crude oil^6^. Embryos were found to accumulate waterborne PAHs dissolved from oil, with toxicity increasing as a mixture enriched with 2-ringed compounds (i.e., naphthalenes) shifted with weathering, to one dominated by 3-ringed (tricyclic) subfamilies (fluorenes, dibenzothiophenes, and phenanthrenes)^9^,^10^. Subsequent research in aquatic systems in particular has revealed distinct activities for virtually all PAH subfamilies, and even individual compounds within subfamilies. However, mechanistic studies of single compounds often use exposure concentrations that are much higher than those observed in contaminated habitats. More work is needed to understand environmentally relevant complex mixtures.

Studies in zebrafish embryos have demonstrated cardiotoxic effects of many single PAH compounds and PAH mixtures, but also revealed a minimal binary division of mechanisms. PAHs that are strong carcinogens are potent agonists of the aryl hydrocarbon receptor (AHR), inducing their own metabolism and carcinogenic activation by cytochrome P4501A^8^. In general, these PAHs are also cardiotoxic to developing fish embryos at relatively high concentrations. Similar to dioxins and polychlorinated biphenyls^11^,^12^, these PAHs inappropriately activate the AHR in developing cardiomyocytes, leading to primary defects in cardiac morphogenesis (poor chamber looping and reduced cardiomyocyte proliferation) followed by secondary functional defects. This form of toxicity is entirely dependent on the AHR and is prevented by AHR gene knockdown. This has been demonstrated, for example, for 4- and 6-ring compounds such as benz(a)anthracene and benzo(a)pyrene^13^,^14^,^15^,^16^, and a C4-alkylated 3-ring compound, retene^17^. In contrast, exposure to either complex PAH mixtures derived from crude oil or single tricyclic compounds that dominate in these mixtures leads to cardiac function defects, followed by secondary morphological defects. Indeed, embryos of many fish species exposed to crude oil-derived PAHs show functional abnormalities that include bradycardia and arrhythmias characteristic of atrioventricular conduction block as well as reduced ventricular contractility^18^,^19^,^20^,^21^,^22^. Importantly, the cardiotoxicity of both crude oil and single non-alkylated tricyclic PAHs occurs without activation of the AHR in cardiomyocytes^18^,^19^,^20^,^21^,^22^, and is not prevented by AHR gene knockdown^11^. Moreover, the cardiotoxicity of crude oil is exacerbated by knockdown of *cyp1a*^23^, indicating that CYP1A-mediated metabolism of crude oil-derived PAHs is protective. Fractionation-based toxicity assays also linked the developmental cardiotoxicity of petroleum products to tricyclic PAH families^15^, as well as a limited number of studies in adult zebrafish [e.g., ref. 24]. Collectively, this work supports the existence of an AHR-independent mechanism by which crude oil-derived PAHs induce cardiac arrhythmia and reduce cardiomyocyte contractility.

We have recently demonstrated that complex PAH mixtures from crude oil affect excitation-contraction (EC) coupling in fish hearts^25^. EC coupling is the physiological process that links electrical excitation with contraction in a cardiomyocyte^26^,^27^. In fishes of the family Scombridae (e.g. mackerels, tunas^28^,^29^), as in mammals^26^, EC coupling begins when an action potential (AP) depolarizes the surface membrane of the cardiomyocyte, and opens voltage-gated ion channels, allowing calcium (Ca^2+^) entry into the cell through L-type Ca^2+^ channels (I_CaL_). This extracellular Ca^2+^ influx triggers the release of additional Ca^2+^ from the internal Ca^2+^ stores of the sarcoplasmic reticulum (SR) via a process termed ‘Ca^2+^-induced Ca^2+^-release’ (CICR). The consequent systolic Ca^2+^ transient, which activates the contractile machinery within the heart muscle cells, is the spatial and temporal sum of such local Ca^2+^ releases^30^,^31^. Relaxation occurs when Ca^2+^ is returned to resting levels by reuptake into the SR via the SR Ca^2+^ ATPase (SERCA) and extrusion from the cell via the sodium calcium exchanger (NCX). Critical for AP repolarization are the opening and closing of voltage-gated sodium (Na^+^), Ca^2+^ and potassium (K^+^) channels, which renew the EC coupling process at every heartbeat. Our earlier work^25^ revealed crude oils disrupt these EC coupling pathways in scombrid fish cardiomyocytes, which explains the bradycardia and arrhythmia previously observed in the whole-heart.

Despite evidence that the tricyclic PAH fraction causes the crude oil heart failure syndrome in developing fish^20^, a direct link between an individual tricyclic PAH and the disruption of EC coupling has not been established. The present work set out to define the molecular moiety(s) of cardiotoxic PAHs from crude oil in three scombrid fishes: the Pacific mackerel (*Scomber japonicas*), the yellowfin tuna (*Thunnus albacares*) and the Pacific bluefin tuna (*Thunnis orientalis*). We then reveal the mechanism for this disruption in atrial and ventricular myocytes from these pelagic predators.

## Results

### Disruption of cellular Ca^2+^ dynamics by a single tricyclic PAH

**Figure 1:**
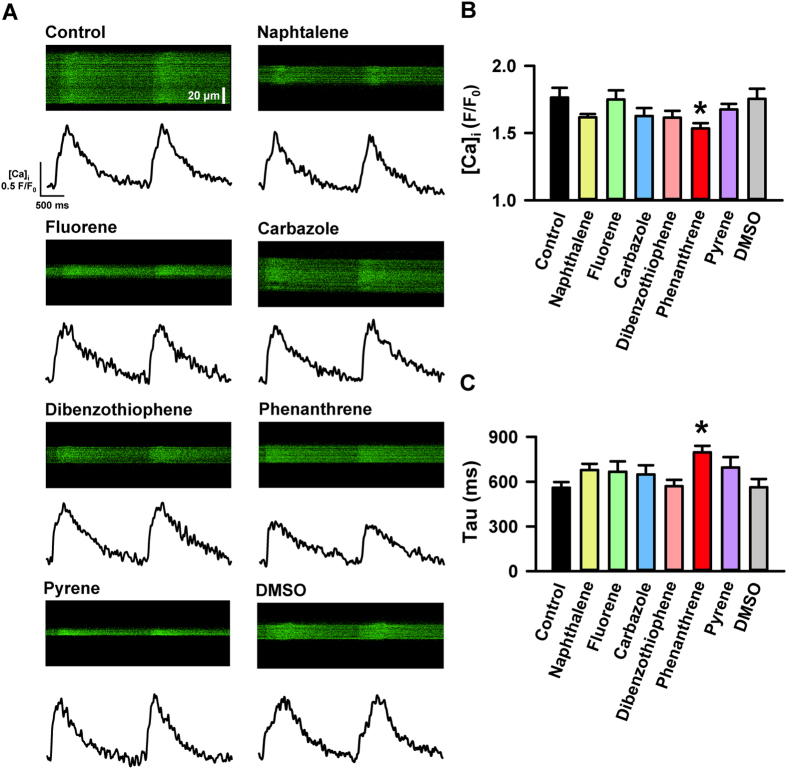
Phenanthrene disrupts Ca^2+^ dynamics in Pacific Mackerel ventricular myocytes. (**A**) Spatial and temporal Ca^2+^ flux in ventricular cardiomyocytes from Pacific mackerel incubated for >30 min with phenanthrene (5 μM), fluorene (5 μM), dibenzothiophene (5 μM), carbazole (5 μM), naphthalene (5 μM), pyrene (5 μM), a DMSO control (1/1000) or untreated (control). Chemical formulae for each moiety are given in Supplementary Fig. S1. Shown here are representative raw confocal transverse line scans across the width of single myocytes loaded with the AM form of the calcium-sensitive dye (Fluo-4) (top) and the corresponding Ca^2+^ transient to indicate the inhibitory effects of individual PAHs on the temporal and spatial characteristics of Ca^2+^ dynamics (bottom). (**B**) Reduction in the mean amplitude of the Ca^2+^ transients expressed as peak fluorescence divided by baseline fluorescence (F/F_0_). (**C**) Increase in the time constant of Ca^2+^ transients decay (tau, time to decay to 37% of its peak). Data are means ± SEM of control (n = 39, N = 10), phenanthrene (n = 42, N = 4), fluorene (n = 21, N = 2), dibenzothiophene (n = 25, N = 3), carbazole (n = 21, N = 2), naphthalene (n = 28, N = 4), pyrene (n = 23, N = 2) and DMSO (n = 22, N = 5). *Indicates significant difference (P < 0.05, one-way ANOVA).

Six PAHs found in crude oil (naphthalene, fluorene, dibenzothiophene, carbazole, phenanthrene and pyrene; Supplementary Fig. S1) were applied individually to ventricular cardiomyocytes isolated from the Pacific mackerel, and Ca^2+^ dynamics were assessed using confocal microscopy. Figure 1A shows the effects of each PAH on the spatial and temporal properties of cellular Ca^2+^ transients recorded using a calcium-sensitive dye, Fluo-4. Cellular Ca^2+^ dynamics were unaffected by PAHs with two benzene rings fused to thiophene and pyrrole rings. By contrast, the 3-ringed PAH, phenanthrene, significantly decreased the Ca^2+^ transient amplitude and slowed the decay (Fig. 1B and C). Changes in Ca^2+^ flux at the single myocyte level culminate in reduced strength and rate of contraction of the whole heart^32^ and could underlie the known reduction in cardiac output in embryonic vertebrate hearts following exposure to crude oil and PAHs. Therefore, we next focused on elucidating the precise mechanism by which phenanthrene altered Ca^2+^ cycling using a combination of confocal Ca^2+^ imaging and electrophysiology in both atrial and ventricular cardiomyocytes.

### Intra- and extracellular mechanisms for altered Ca^2+^ transients in tuna myocytes

**Figure 2:**
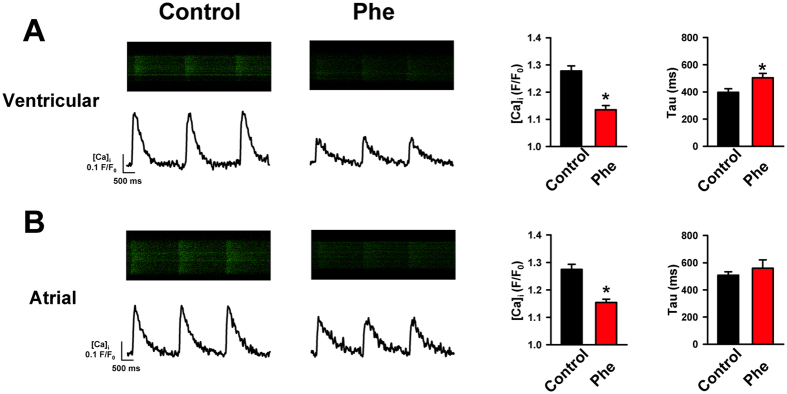
Phenanthrene inhibits intracellular Ca^2+^ flux in bluefin tuna cardiomyocytes. In ventricular (**A**) and atrial (**B**) cardiomyocytes, the amplitude (F/F_0_) and decay (Tau, ms) of Ca^2+^ transients were depressed by phenanthrene (Phe, 5 μM) relative to control (time-matched untreated). Figure shows raw confocal line scan images and the corresponding time courses to the left and the mean data ± SEM on the right (Control n = 19, N = 4; Phe n = 14, N = 3 for ventricle; Control n = 38, N = 8; Phe n = 16, N = 4 for atria). *Indicates significant difference, (P < 0.05, Student’s t-test).

**Figure 3:**
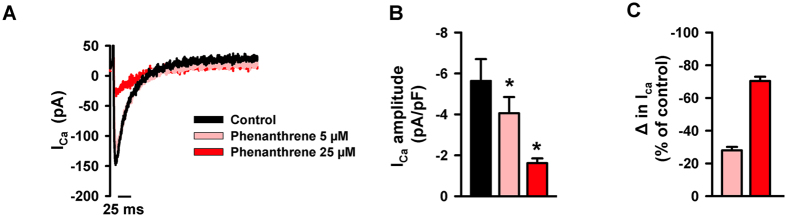
Phenanthrene decreases extracellular Ca^2+^ influx via the L-type Ca^2+^ current, I_CaL_ in bluefin tuna ventricular myocytes. (**A**) L-type Ca^2+^ channel current (I_CaL_) density recorded from bluefin tuna ventricular myocytes via whole-cell voltage clamp in control condition (black trace) and with ascending concentrations of phenanthrene (pink/red traces) subsequently perfused over the same cell. (**B**) Mean data ± SEM (n = 6, N = 2) I_CaL_ amplitude in control (black bar) and with an increasing concentrations of phenanthrene (pink bar, 5 μM; red bar, 25 μM). (**C**) Change in I_CaL_ amplitude (expressed as a percentage of control) with ascending concentrations of phenanthrene (pink bar, 5 μM; red bar, 25 μM). Similar inhibitory effects were found in two atrial myocytes (not shown). *Indicates significant difference (P < 0.05, Student’s t-test or one-way ANOVA).

Similar to Pacific mackerel, phenanthrene (5 μM) impaired Ca^2+^ transient amplitude and decay rate in both ventricular and atrial cardiomyocytes from Pacific bluefin tuna (Fig. 2A and B) and yellowfin tuna (Supplementary Fig. S2). Thus, in three ecologically and commercially important pelagic species, the toxic effects of a single tricyclic PAH on cardiac cellular dynamics mirror those observed in response to more chemically complex crude oil^25^. A reduction in Ca^2+^ transient amplitude could be due to reduced extracellular Ca^2+^ influx (I_CaL_) through L-Type Ca^2+^ channels and/or a smaller Ca^2+^ release from SR internal Ca^2+^ stores. To clarify this point, we first used whole-cell voltage-clamp to investigate extracellular Ca^2+^ influx via I_CaL_. We found that phenanthrene decreased the amplitude of I_CaL_ (Fig. 3A–C). I_CaL_ is the major source of extracellular Ca^2+^ entry across the sarcolemma during EC coupling in all vertebrate hearts, including tunas and mackerel^26^,^29^,^33^. I_CaL_ contributes substantially to the amplitude of the Ca^2+^ transient and is also the major extracellular Ca^2+^ trigger for CICR from intracellular SR Ca^2+^ stores^26^. Therefore, phenanthrene’s action on the Ca^2+^ transient could be both direct, by reducing extracellular Ca^2+^ influx, and indirect by reducing the cytosolic Ca^2+^ available to trigger Ca^2+^ release from (depleted) internal SR stores.

**Figure 4:**
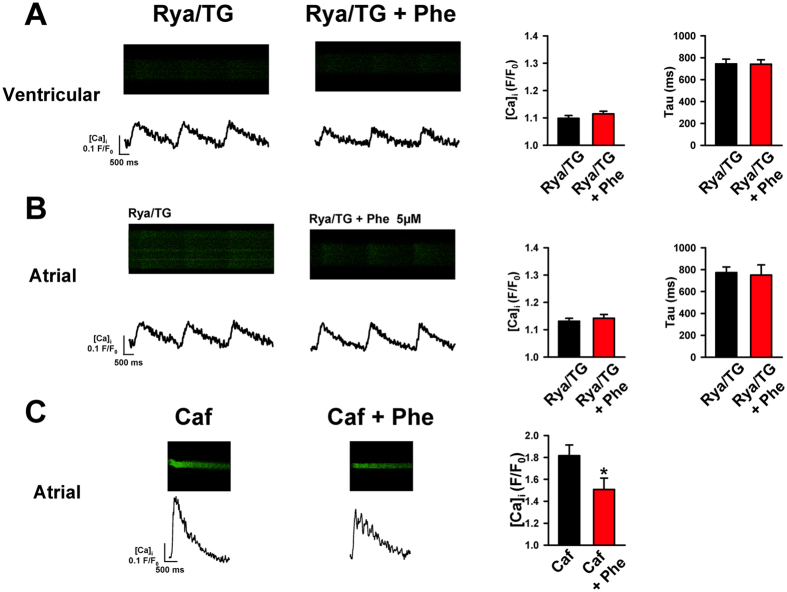
Role of sarcoplasmic reticulum function in phenanthrene toxicity in bluefin tuna cardiomyocytes. Inhibiting Ca^2+^ cycling through the sarcoplasmic reticulum (SR) with ryanodine (Rya, 5 uM) and thapsigargin (Tg, 2 uM) depressed the amplitude and decay rate of the Ca^2+^ transient in ventricular (**A**) and atrial (**B**) cardiomyocytes. Importantly, it also abolished the effect of phenanthrene (right panel). Mean data are ± SEM for ventricular (Rya/Tg, n = 13, N = 3; Rya/Tg + Phe, n = 20, N = 2) and atrial cardiomyocytes (Rya/Tg, n = 15, N = 3; Rya/Tg + Phe, n = 12, N = 2), are shown to the right. (**C**) Phenanthrene decreased the Ca^2+^ content of the sarcoplasmic reticulum. Ca^2+^ transients recorded from bluefin tuna atrial cardiomyocytes after a rapid caffeine puff (Caf, 20 mM) in the presence and absence of phenanthrene pre-treatment. Raw confocal line scan images and the corresponding transient time courses are shown. Mean data ± SEM showing Caffeine-induced Ca^2+^ transient amplitude (F/F0) in controls (Caf; n = 9, N = 2) and phenanthrene pre-exposed (Caf + Phe; n = 9, N = 2) are shown to the right. *Indicates significant difference, (P < 0.05, Student’s t-test).

To examine these interactions, we next exposed atrial and ventricular cardiomyocytes to pharmacological inhibitors of SR Ca^2+^ release (5 μM ryanodine to inhibit the SR Ca^2+^ release channel, ryanodine receptor) and SR Ca^2+^ uptake (2 μM thapsigargin to inhibit SERCA) for 30 minutes before exposing to phenanthrene. As anticipated from earlier work^28^, SR inhibition decreased the amplitude and the rate of decay of the bluefin tuna Ca^2+^ transient (Fig. 4). This confirms that the hearts of these active predators utilize SR Ca^2+^ stores during EC coupling, unlike many sedentary species of fish^29^. Pharmacological pre-blockade of SR Ca^2+^ cycling eliminated the effects of phenanthrene on the amplitude and the decay of the cytosolic Ca^2+^ transient (Fig. 4A and B). To further investigate the role of SR internal Ca^2+^ stores, we exposed atrial myocytes to a puff of caffeine (20 mM), which causes the ryanodine receptors to open and empty SR Ca^2+^ into the cytosol. We found SR Ca^2+^ content was significantly decreased in myocytes exposed to phenanthrene (Fig. 4C), indicating a diminished SR Ca^2+^ load. Taken together, these results suggest that phenanthrene slows the decay of the transient by limiting the reuptake of Ca^2+^ into the SR via SERCA. The smaller Ca^2+^ transient amplitude in the presence of phenanthrene could be caused by a the reduction in the I_Ca_ trigger for SR release, direct effects on SR Ca^2+^ release through ryanodine receptors, or both.

### *I*_*Kr*_ blockade and AP prolongation are the basis for cardiac arrhythmogenesis

**Figure 5:**
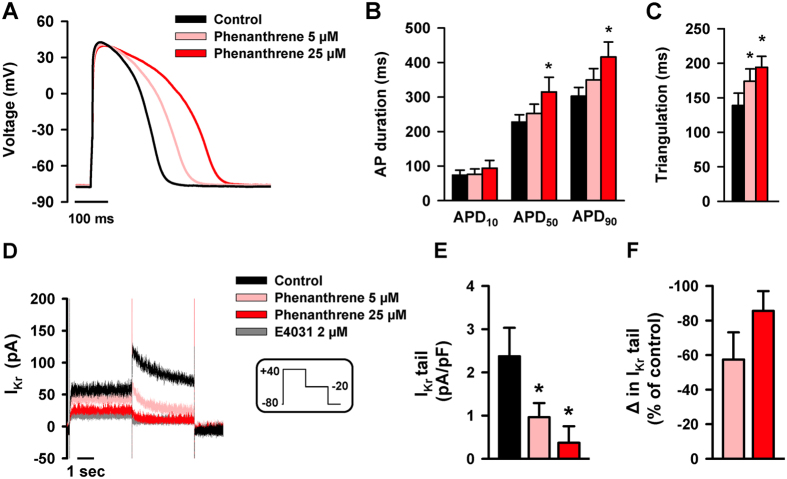
Phenanthrene disrupts electrical activity of the bluefin tuna ventricular myocytes. (**A**) Representative action potentials (APs) recorded with current-clamp in a ventricular myocyte under control conditions (black) and with an increasing concentration of phenanthrene (pink/red traces). (**B**) Means ± SEM (n = 9, N = 5) of action potential duration (APD) at 10, 50 and 90% repolarization and (**C**) AP triangulation (calculated as APD_90_ − APD_30_) before and after phenanthrene exposure. (**D**) Reduction in the delayed rectifier K^+^ channel current (I_Kr_) density recorded with voltage-clamp under control conditions (black) and with an increasing concentration of phenanthrene (pink/red traces) or with the I_Kr_ specific blocker E4031 (gray trace). (**E**) Means ± SEM of peak I_Kr_ tail in control (black bar) and with an increasing concentration of phenanthrene (pink/red bars, n = 7, N = 3). (**F**) Change in I_Kr_ tail (expressed as a percentage of control) in response to phenanthrene. *Indicates significant difference (P < 0.05, one-way ANOVA).

EC coupling is initiated by the AP. To assess whether phenanthrene alters this essential electrical property of excitable cardiomyocytes, we used whole-cell current-clamp to record APs prior to and during application of ascending concentrations of phenanthrene. Phenanthrene caused a rapid (<1 min) and significant prolongation of AP duration (APD) associated with a dose-dependent increase in triangulation (APD_90_-APD_30_) (Fig. 5). Such proarrhythmic responses have been implicated in atrial and ventricular fibrillation and sudden cardiac death in a number of species^34^,^35^.

The delayed rectifier K^+^ channel current (I_Kr_), is the key ion current responsible for AP repolarization. To determine whether the observed AP prolongation was due to an inhibition of outward K^+^ conductance, we evaluated the effects of phenanthrene on I_Kr_. As shown in Fig. 5D–F, I_Kr_ was significantly reduced by ascending concentrations of phenanthrene. In addition to explaining the prolongation of the AP, this mechanism is consistent with the phenanthrene- and crude oil-induced arrhythmogenesis and atrioventricular conduction block previously reported in other fish species^18^,^20^,^22^. Phenanthrene did not affect the cardiomyocyte resting membrane potential, AP amplitude or the rate of rise of the AP (i.e., AP traces in Fig. 5A and Supplemental Fig. S3).

## Discussion

Our data demonstrate that impairment of EC coupling in cardiomyocytes by phenanthrene is a key determinant of cardiotoxicity from crude oil. This disruption of excitable cell pathways now establishes a mechanism for crude oil cardiotoxicity that has proven elusive for decades. First, phenanthrene affects membrane excitability by prolonging AP duration by inhibiting K^+^ efflux from the cardiomyocyte via I_Kr_. Second, phenanthrene reduces Ca^2+^ influx into the cell by reducing I_CaL_. This reduces myofilament activation and thus myocyte contractility. Third, inhibition of I_CaL_ has a knock-on effect as it reduces Ca^2+^ release from the SR, which further impairs cardiac contractility. Combined, these disruptions to EC coupling in the myocyte can lead to contractile failure and abnormal contractile rhythm. Our findings are in agreement with previous studies showing that SERCA function is altered in phenanthrene-exposed zebrafish^36^ and miR-133a, a microRNA known to regulate hypertrophy, is reduced in rats with phenanthrene-induced cardiac hypertrophy^37^. Although some of the larger 4- and 6-ring PAHs have also been shown to alter expression of genes involved in SR calcium handling (e.g., *atp2a2*/SERCA2 and *ryr2*/ryanodine receptor), these effects must be downstream of AHR activation^38^,^39^.

By applying single PAHs directly to isolated cardiomyocytes, we have identified AHR-independent cellular mechanisms that likely underpin the whole-heart cardiotoxicity phenotypes previously observed in oil-exposed fish embryos. Because of the very small size of the embryonic fish heart (e.g., roughly 300 cardiomyocytes in zebrafish; ref. 11), its limited capacity for CYP1A-mediated detoxification, and its close proximity to PAH uptake across the embryonic epidermis, reducing potential first-pass metabolic protection by CYP1A in other tissues, isolated cardiomyocytes from relative mature fish are a good proxy for the intact hearts of developing fish embryos. Prior to liver formation and the associated capacity for metabolic detoxification, fish embryos bioconcentrate tricyclic PAHs to very high tissue concentrations – i.e., in the low parts-per-million or micromolar range (*e.g.* refs 18 and 40). For example, parent phenanthrene levels are higher than the alkylated homologs in Alaskan crude oil at the early stages of weathering. Pacific herring embryos exposed to this oil accumulated parent phenanthrene to tissue concentrations as high as 2.5 μM (450 parts per billion) and showed pronounced cardiac arrhythmia^18^. Thus, the exposure concentrations used here for isolated cardiomyocytes correspond to the tissue levels that produce heart form and function defects in whole embryos. Additional contributions from AHR-dependent pathways^17^,^21^, if any, would increase the toxic potency of complex PAH mixtures.

Our findings will help refine natural resource injury assessments for future oil spills in fish spawning habitats. As spilled oil weathers it becomes relatively enriched in phenanthrenes, thereby explaining why weathered oil (by mass) is proportionally more toxic to the fish heart. Our results also help explain why certain geologically distinct and phenanthrene-enriched crude oils have more significant cardiotoxic impacts, although we acknowledge that many PAHs and mixtures of PAHs exert their toxicity via multiple different mechanisms. This new insight may simplify the interpretation of water samples collected during oil spills for natural resource injury assessments. Future assessments should give particular weight to measured and modeled levels of phenanthrenes, in addition to complex PAH mixtures that dynamically shift in space and time throughout regions impacted by spilled oil.

Lastly, given that phenanthrenes are a near-ubiquitous component of complex environmental PAH mixtures in the oceans and the atmosphere, our findings have implications for humans that extend well beyond the Deepwater Horizon spill. A major area of public health research over the last decade has focused on the acute cardiac impacts of urban air pollution, but the precise etiology of these effects remains elusive^41^. The pro-arrhythmic actions of phenanthrene may be particularly relevant in this regard. In humans, I_Kr_ is generated by the voltage-gated potassium channels hERG1 or hERG2^42^. Blockade of the hERG channel can lead to life-threatening arrhythmias, making this channel an important therapeutic target. Genetic and chemical studies in zebrafish indicate that the function and pharmacology of these channels are nearly identical across vertebrates^43^,^44^,^45^. Consequently, we suggest that atmospheric phenanthrene should be a concern for human cardiology, particularly due to its high abundance in urban air^46^ and its rapid absorption into the bloodstream after inhalation^47^. This study should raise global interests in this important environmental pollutant given the prevalence of petroleum and PAHs in our environment.

## Conclusion

We have identified a cardio-toxicant compound prominent in crude oil, and shown how it alters cardiac force and cardiac rhythm in pelagic fish. This provides a new framework for evaluating the effects of PAHs on cardiac function that we believe can be extended across a wide range of vertebrates, including humans. Reducing future releases of this pro-arrhythmic chemical should substantively benefit human and ecological health.

## Methods

### Fish origin and care

Mackerel (fish mass = 0.69 ± 0.14 kg, heart mass = 1.3 ± 0.3 g, mean ± SEM, N = 11), bluefin tuna (fish mass = 14.1 ± 1.0 kg, heart mass = 52.9 ± 4.8 g, mean ± SEM, N = 16) and yellowfin tuna (fish mass = 12.2 ± 2.0 kg, heart mass = 30.9 ± 37 g, mean ± SEM, N = 6) were captured off San Diego, CA, held aboard the *F/V Shogun* in seawater wells, and then transported by truck to the Tuna Research and Conservation Center (Pacific Grove, CA). Mackerel and tunas were held in a 30-m^3^ and 109-m^3^ tank respectively at 20 ± 1 °C and fed a diet of squid, sardines, and enriched gelatin, as previously described^48^,^49^. Fish were acclimated to 20 °C for at least 4 weeks before experimentation. Individual cardiomyocytes were isolated using protocols previously described in detail^28^. All procedures were in accordance with Stanford University Institutional Animal Care and Use Committee protocols. All experimental protocols were approved by the Stanford University Animal Care and Use Committee.

### Chemicals

All solutions were prepared using ultrapure water supplied by a Milli-Q system (Millipore, USA). Chemicals were reagent grade and purchased from Sigma (St. Louis, MO) except for TTX (Tocris, UK), ryanodine (Ascent Scientific, MA), Fluo-4 (Molecular Probes, NY) and E-4031 (Enzo Life Sciences, NY). Purities for all PAHs were at least 99%. Stock PAH solutions were constituted in dimethyl sulfoxide (tissue culture grade, Sigma) at 5 mM.

### Physiological solutions

The composition of the extracellular physiological solution (Ringer) used for both Ca^2+^ imaging and electrophysiology was based on previous work^33^ and contained (in mM) 150 NaCl, 5.4 KCl, 1.5 MgCl_2_, 3.2 CaCl_2_, 10 glucose and 10 HEPES, with pH set to 7.7 with NaOH. To avoid overlapping ion currents when recording I_Kr_ and I_CaL_, this solution was modified. For I_CaL_ measurement, KCl was replaced by CsCl to inhibit K^+^ channels. For I_Kr_ recording, tetrodotoxin (TTX, 0.5 μM), nifedipine (10 μM), and glibenclamide (10 μM) were included in the Ringer solution to inhibit Na^+^, Ca^2+^, and ATP-sensitive K^+^ channels, respectively.

Pipette solutions were optimized for each electrophysiological recording. For AP measurement, the pipette solution contained (in mM): 10 NaCl, 140 KCl, 5 MgATP, 0.025 EGTA, 1 MgCl_2_, and 10 HEPES, pH adjusted to 7.2 with KOH. For I_Ca_ measurement, the pipette solution contained (in mM) 130 CsCl, 15 TEA-Cl, 5 MgATP, 1 MgCl_2_, 5 Na_2_-phosphocreatine, 0.025 EGTA, 10 HEPES, and 0.03 Na_2_GTP, pH adjusted to 7.2 with CsOH. CsCl and TEA-Cl were included to inhibit K^+^ currents. The low concentration of EGTA was included to approximate physiological Ca^2+^ buffering capacity^50^. For I_Kr_, the pipette solution contained (in mM): 10 NaCl, 140 KCl, 5 MgATP, 5 EGTA, 1 MgCl_2_, and 10 HEPES, pH adjusted to 7.2 with KOH. The high concentration of EGTA was included to block Na^+^ -Ca^2+^ exchanger current.

### Intracellular [Ca^2+^] measurements

Confocal Ca^2+^ imaging was performed using a laser-scanning unit attached to an Olympus inverted microscope. Control and PAH-exposed myocytes were loaded with 4 μM Fluo-4 AM (Molecular Probes) for 20 min, washed via dilution to de-esterify and then perfused with standard Ringer solution. The dye was excited at 488 nm and fluorescence measured at >500 nm. Transverse line scans were acquired at 5 ms intervals. Cells were electrically stimulated at 0.5 Hz via extracellular electrodes. Batches of myocytes were incubated with 5 μM of PAH for at least 1 hour. Control experiments were performed on time-matched untreated cells. Some cells from both control and PAH-exposure groups were incubated with SR inhibitors (5 μM ryanodine and 2 μM thapsigargin) for at least 30 minutes prior to imaging. In some experiments, a caffeine pulse (20 mM) was applied via a home-built rapid solution system. All line scan images are presented as original raw fluorescence (F). Background fluorescence (F_0_) was measured in each cell in a region that did not have localized or transient fluorescent elevation.

### Electrophysiological recordings

Electrophysiological data was recorded as previously described^51^. Briefly, at the beginning of each trial, myocytes were allowed to settle for ~10 minutes in the recording chamber, and then perfused with control external solution. Membrane potentials and currents were recorded from each myocyte in whole-cell mode under baseline (control) conditions, and again at increasing concentrations of phenanthrene in the extracellular solution. Stimuli used to elicit ion currents and APs are provided in the figure legends. Briefly, the L-type Ca^2+^ channel current (I_CaL_) was elicited by a pulse to 0 mV (the approximate peak of the current-voltage relationship in cardiomyocytes) after a pre-pulse to −40 mV to inactivate Na^+^ current. I_CaL_ was measured as the difference between peak and the end of pulse current. Trains of depolarizing pulses were applied at 0.2 Hz. Action potentials (APs) were evoked using 10 ms sub-threshold current steps at a frequency of 0.5 Hz. The delayed rectifier K^+^ current (I_Kr_) was measured using an established protocol adapted from previous studies on Pacific bluefin tuna^52^ and rainbow trout^53^. I_Kr_ was activated by a pre-pulse to +40 mV (to fully activate K^+^ channels) and measured as the tail current at −20 mV, the maximum tail current in tuna myocytes^53^. To separate rapid K^+^ current (I_Kr_), tail I_K_ amplitude was measured as the current sensitive to a specific I_Kr_ inhibitor (2 μM E-4031). Trains of depolarizing pulses were applied 0.2 Hz every 20 seconds. Data was recorded via a Digidata 1322 A A/D converter (Axon Instruments, CA) controlled by an Axopatch 200B (Axon Instruments, CA) amplifier running pClamp software (Axon Instruments, CA). Signals were filtered at 1–10 kHz using an 8-pole Bessel low pass filter before digitization at 10–20 kHz and storage. Patch pipette resistance was typically 1.5–3 MΩ when filled with intracellular solution (below). Cell membrane capacitance was measured using the “membrane test module” in Clampex (fitting the decay of the capacitance current recorded during a 10 mV depolarizing pulse from a holding potential of −80 mV).

### Data analysis

Ca^2+^ transients from confocal experiments were analyzed using Image J, Clampfit (Axon Instruments, CA), and Origin (OriginLab Corporation, MA). At least three traces at steady state were analyzed and averaged. The decay of the Ca^2+^ transient was fitted with a single exponential to calculate Tau of decay (i.e., time to decrease to 37% of the peak amplitude). The amplitude of the Ca^2+^ transient is defined as peak increase and basal Ca^2+^ (F/F_0_)^54^. Electrophysiological data were analyzed using Clampfit and Origin software. All AP parameters were stable over the time of recording (<10 min) in controls. Currents are expressed as current density (pA/pF). I_CaL_ was measured as the difference between the peak inward current and the current at the end of the depolarizing pulse. I_Kr_ amplitude was measured as the current sensitive to E-4031.

### Statistics

Data are presented as mean ± SEM Statistical analysis was performed using SigmaStat software (Systat Software, CA). Electrophysiological data with a confirmed normal distribution and equal variance were analyzed using One Way Repeated Measures Analysis of Variance or Friedman Repeated Measures Analysis of Variance on Ranks followed by a Student-Newman-Keuls test for post hoc analysis. For confocal data, unpaired Student’s t-test were used within the same species of tuna, one-way ANOVAs were used to test for the effect of PAHS in mackerel, and two-way ANOVAs were used to determine interactions between species, SR inhibition and PAHs. P < 0.05 was considered significant.

## Additional Information

**How to cite this article**: Brette, F. *et al*. A Novel Cardiotoxic Mechanism for a Pervasive Global Pollutant. *Sci. Rep.* **7**, 41476; doi: 10.1038/srep41476 (2017).

**Publisher's note:** Springer Nature remains neutral with regard to jurisdictional claims in published maps and institutional affiliations.

## Supplementary Material

Supplementary Data
