# Supplementary material for: A Novel Cardiotoxic Mechanism for a Pervasive Global Pollutant
Source: Sci Rep. 2017 Jan 31;7:41476. doi: 10.1038/srep41476 (PMC5282528; doi:10.1038/srep41476)
Supplement: Supplementary Data [file srep41476-s1.doc]

**Supplementary Materials:**

**A Novel Cardiotoxic Mechanism for a Pervasive Global Pollutant**

Fabien Brette^*^, Holly A. Shiels^*^, Gina L.J. Galli, Caroline Cros, John P. Incardona, Nathaniel L. Scholz, Barbara A. Block^#^


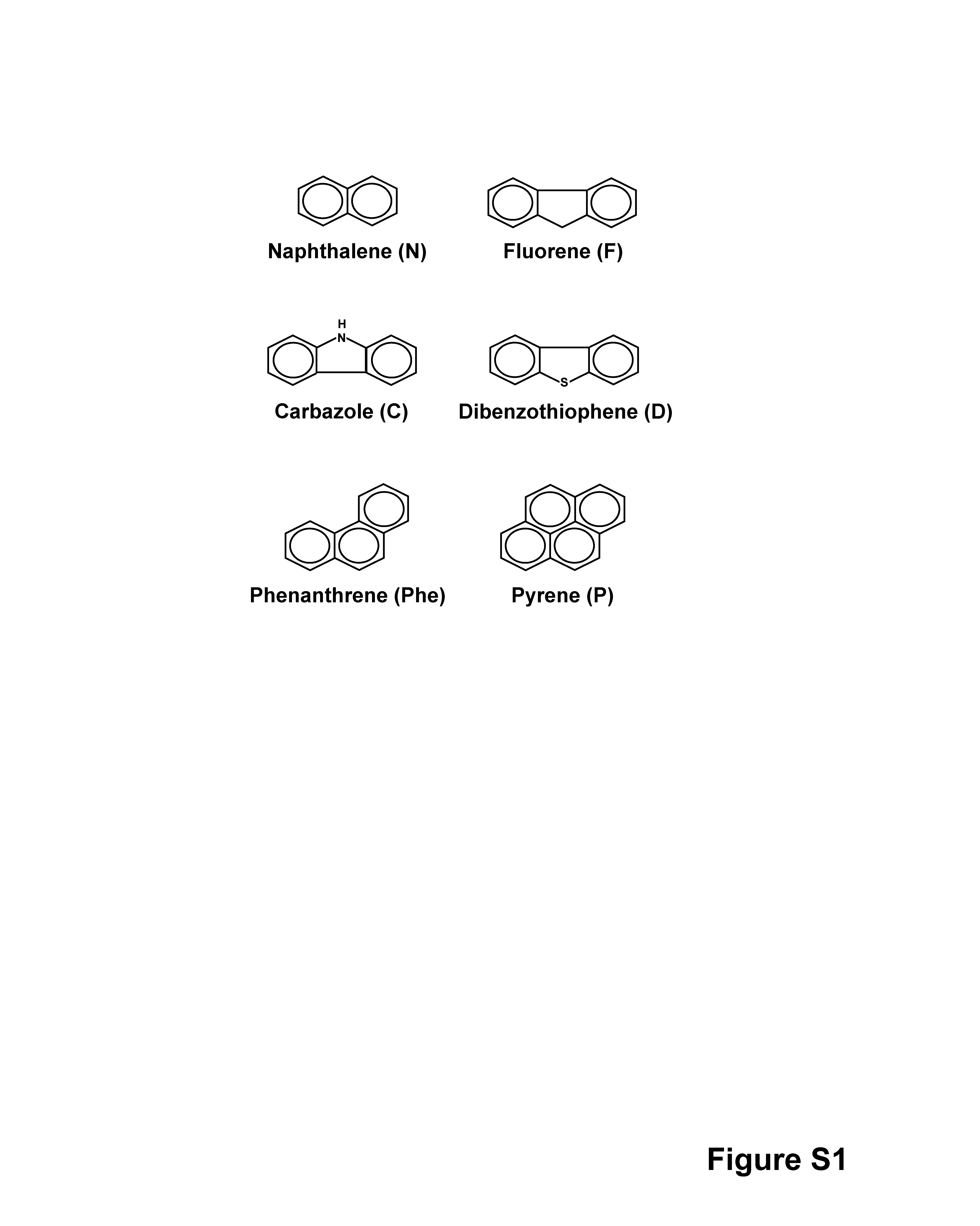


**Figure S1: Chemical structures for the polycyclic aromatic compounds used in this study.**

These include polycyclic aromatic hydrocarbons (PAHs; naphthalene, fluorene, phenanthrene, and pyrene) and closely related S- and N-containing heterocycles (dibenzothiophene and carbazole, respectively).


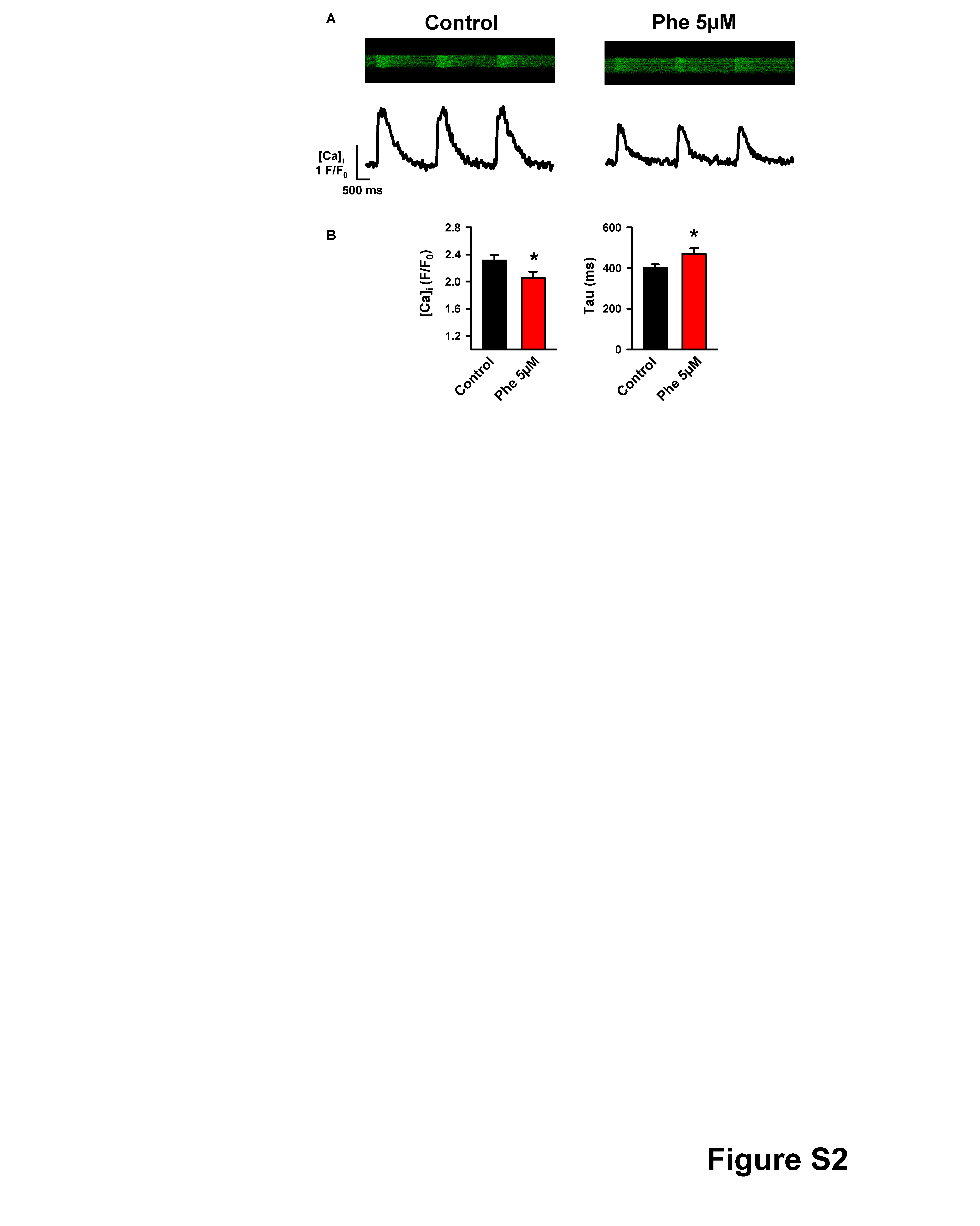


**S2: Phenanthrene disrupts Ca^2+^ transients in ventricular myocytes from yellowfin tuna.**

(*A*) The effect of phenanthrene (Phe, 5 µM) on the raw confocal line scan images and corresponding Ca^2+^ transients. (*B*) Mean data ± SEM showing the reduction in Ca^2+^ transient amplitude (F/F_0_) and slowing of the decay of Ca^2+^ transients (Tau) with phenanthrene treatment (red bars) compared with control (black bars). Ctl: n = 55, N=5; Phe: n = 37, N=4. * indicates significant difference, Student’s t-test (P < 0.05).


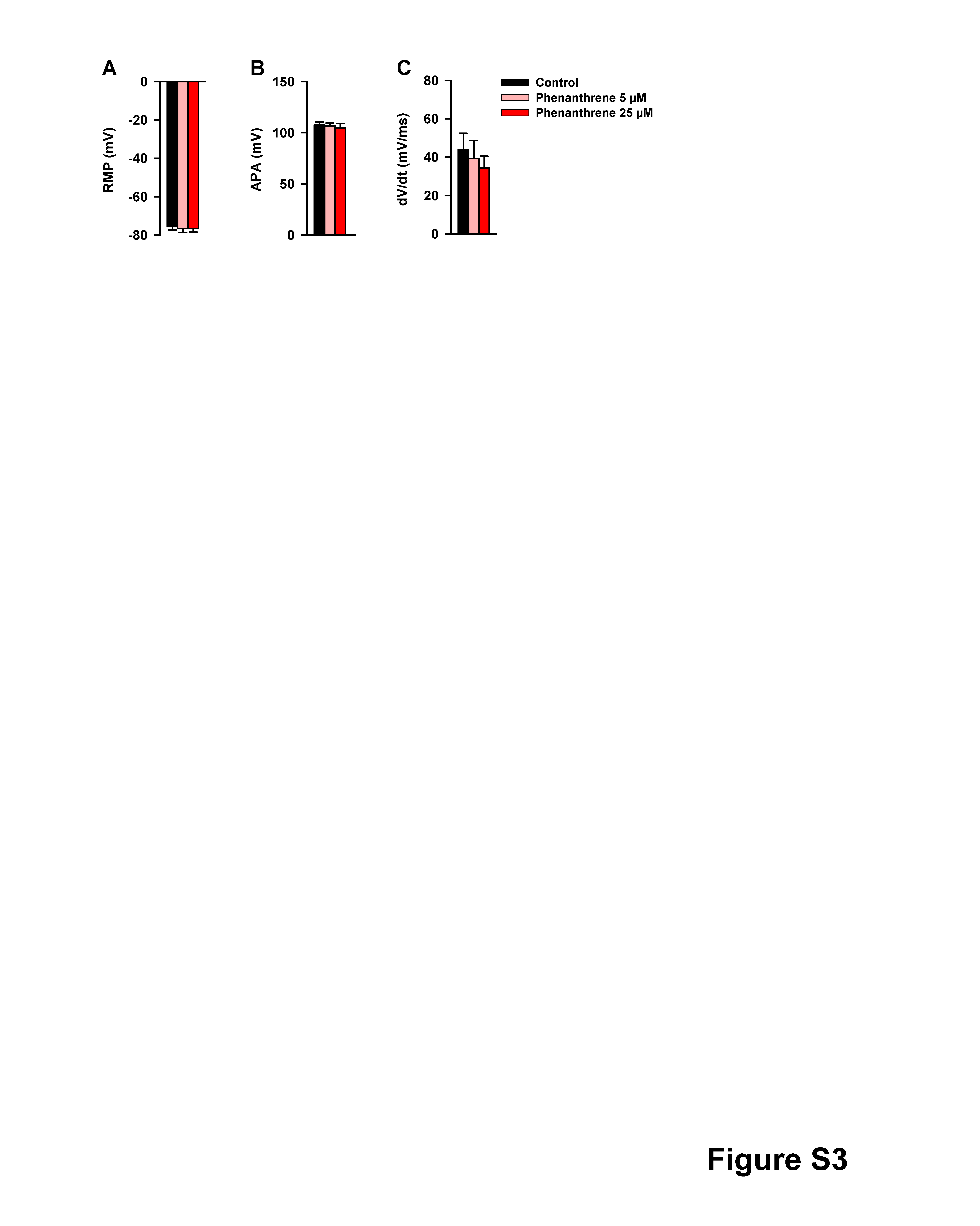


**S3: Phenanthrene does not alter other action potential characteristics in ventricular myocytes from bluefinfin tuna.**

(*A*) Resting membrane potential (RMP), *(B)* action potential amplitude (APA) and *(C)* dV/dt recorded in control condition (black bars) and in ascending concentrations of phenanthrene (red bars). Means ± SEM. Data are from n = 9, N=5.
